# Seasonal Analysis of *Candida auris* Detection Rates at 80 Wastewater Monitoring Sites in the United States, December 2023–November 2024

**DOI:** 10.1111/1758-2229.70376

**Published:** 2026-06-08

**Authors:** Jorge Chavez, Alessandro Zulli, Dustin Duffy, Mariya Campbell, Meghan Lyman, Angela Coulliette‐Salmond, D. Joseph Sexton

**Affiliations:** ^1^ Clinical Environmental Microbiology Branch, National Center for Emerging and Zoonotic Infectious Diseases Centers for Disease Control and Prevention Atlanta Georgia USA; ^2^ Department of Civil and Environmental Engineering Stanford University Stanford California USA; ^3^ Mycotic Diseases Branch, National Center for Emerging and Zoonotic Infectious Diseases Centers for Disease Control and Prevention Atlanta Georgia USA; ^4^ U.S. Public Health Service Rockville Maryland USA

**Keywords:** antimicrobial resistance, *Candida auris*, fungi, public health, seasonal, wastewater

## Abstract

*Candida auris* is an emerging fungal pathogen known to cause outbreaks in healthcare settings that are difficult to control. Here, data from 80 wastewater treatment plants were collected between December 2023 and November 2024 to compare *C. auris* seasonal detection rates across the four meteorological seasons. The mean *C. auris* seasonal detection rate communicated as a percentage (((# of detections per site/# test days per site)/# total sites)*100) was highest in the summer (7%), followed by fall (4%), spring (4%) and winter (2%). A Kruskal–Wallis test identified a significant difference between the seasons (H = 43.6, *p* < 0.0001) and a Dunn's post hoc test with Bonferroni correction identified pairwise differences between summer and each of the other seasons (*p* < 0.05). Statistical significance and 95% confidence intervals (CI) were further assessed by a bootstrapping analysis that also identified a significant difference between summer and all other seasons (*p* < 0.01). Proportionally, 39 (48%) sites had their highest seasonal detection rate in summer, followed by 21 (27%) in fall, 15 (18%) in spring and five (7%) in winter. These results collectively document elevated *C. auris* seasonal detection rates in summer, providing new context to apply *C. auris* wastewater surveillance.

## Graphical abstract

This study documents seasonal differences in *Candida auris (C. auris)* wastewater detection rates in the United States, reporting the highest rates in summer. This work improves the understanding of *C. auris* in wastewater and provides context for applying wastewater‐based epidemiology to inform public health decisions.

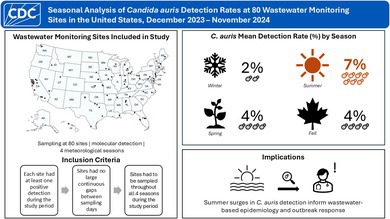

## 1 Introduction

*Candida auris* ( *C. auris* ) is an emerging multidrug‐resistant yeast that spreads easily in healthcare settings and can cause severe infections with high mortality (Jackson et al. 2019). It was designated as an ‘Urgent Threat’ in the 2019 Centers for Disease Control and Prevention (CDC) Antibiotic Resistance Threats Report (Centers for Disease Control and Prevention (CDC) 2019). *C. auris* was first clinically described in 2009 and has since spread around the globe with an increasing spread documented during the COVID‐19 pandemic (Centers for Disease Control and Prevention (CDC) 2019; Lyman et al. 2023). Distinctive properties such as thermotolerance and an ability to heavily colonise skin, contaminate the healthcare environment and persist on surfaces for weeks contribute to the continued spread of *C. auris* (Jackson et al. 2019).

Given the challenges controlling *C. auris* outbreaks in healthcare settings, early detection is important for timely implementation of infection control and prevention practices. For this reason, there is growing interest in incorporating *C. auris* wastewater‐monitoring as an early detection strategy. Reports from Florida, Maryland, Nevada and Utah have demonstrated *C. auris* can be detected in wastewater systems and can sometimes be associated with local clinical or colonisation cases (Chavez et al. 2024; Barber et al. 2023; Nwaubani et al. 2025; Babler et al. 2023). However, there are still knowledge gaps that limit capacity to apply *C. auris* wastewater detections to inform public health response. Specifically, whether a wastewater detection event might be indicative of a recent local clinical case or instead be attributable to baseline *C. auris* wastewater concentrations. For these reasons, it would be helpful to better understand seasonal trends in *C. auris* detection rates.

WastewaterSCAN (WWSCAN) is a research program that monitors infectious diseases, including *C. auris* , through municipal wastewater systems. During the timeframe of this study, it monitored 190 sites across 40 states and in Washington, D.C. within the US (Boehm et al. 2024; Zulli et al. 2024; Wastewater Scan 2024). The program tests for pathogen biomarkers at participating wastewater treatment plants by extracting nucleic acids from the solid phase of wastewater samples year‐round. WWSCAN reports *C. auris* nucleic acid concentrations in units of copies per gram dry weight (cp/g) normalised by pepper mild mottle virus (i.e., marker of human waste) for each sampling day (Zulli et al. 2024; Wastewater Scan 2024; Symonds et al. 2019). The full methods for measuring *C. auris* are provided in Zulli et al. (2024). Using the sample dates available during September 2023 to February 2024, Zulli et al. found that *C. auris* seasonal detection rates from 190 sites were significantly higher in the fall months compared to the winter months. Herein, we expand on that work by presenting an analysis of *C. auris* data over a full calendar year from December 2023 to November 2024 comparing *C. auris* seasonal detection rates across the four meteorological seasons.

## 2 Methods

### 2.1 Site Inclusion Criteria and Data Collection

**FIGURE 1:**
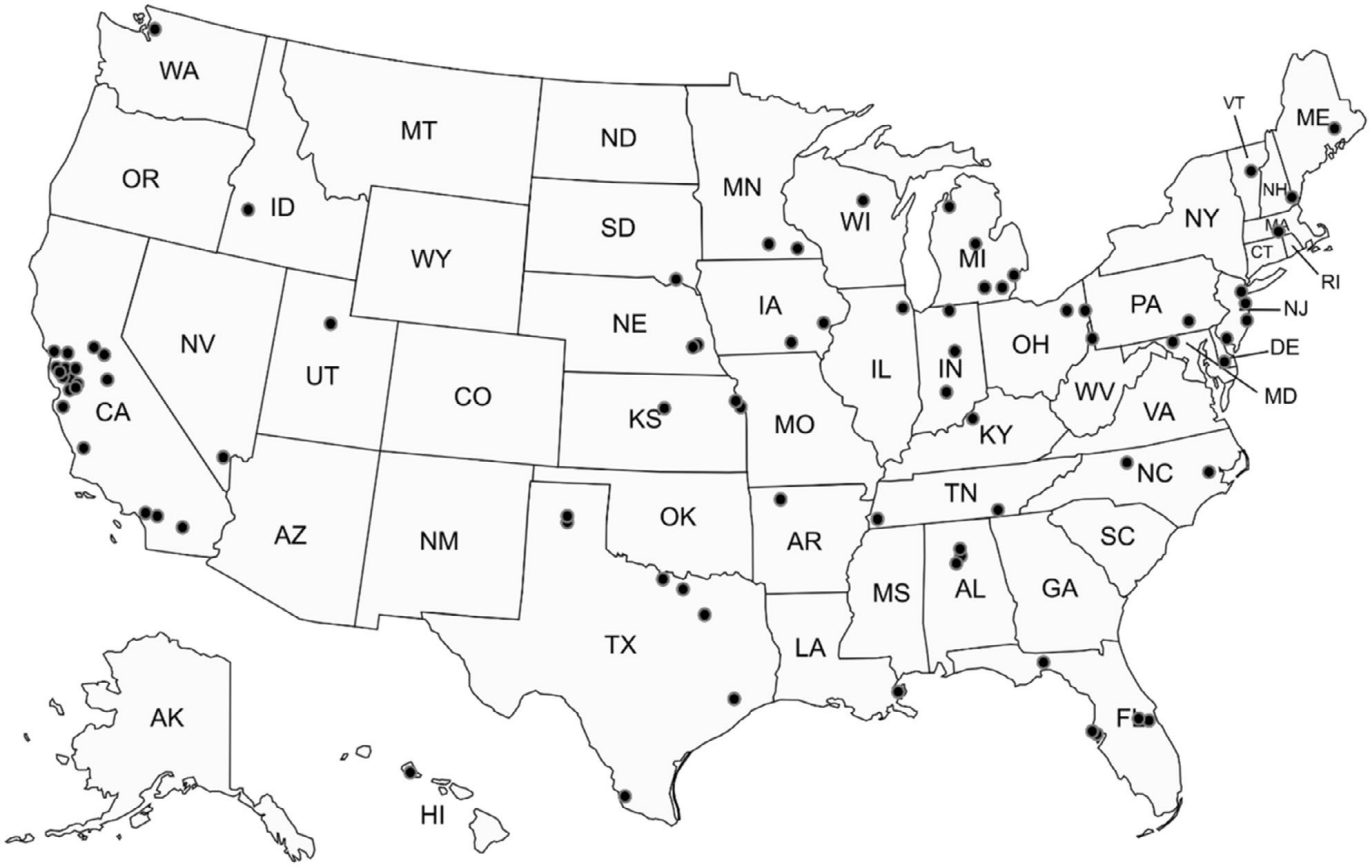
Approximate location of 80 U.S. wastewater treatment plants that met study inclusion criteria, 12/01/2023 to 11/30/2024.

Sample collection and processing were conducted by the WWSCAN research program as described in Zulli et al. (2024). To compare *C. auris* seasonal detection rates across all four meteorological seasons over a 12‐month time frame, a study period was defined 12/01/2023 to 11/30/2024. Although WWSCAN did have *C. auris* monitoring data dating back to September 2023, the study period was chosen since it marked the first full year that was sampled and encompassed all seasons. Only sites with at least one positive detection anytime during the study period were included in the analysis, with a positive being defined as any quantitative result reported as > 0 cp/g. Of the 190 WWSCAN sites, 110 (58%) reported positive *C. auris* detection data during the study period. Of those, 98 reported monitoring data in all four seasons. To further ensure only sites with comprehensive coverage across the 12 months and four seasons were included, a histogram of ‘longest gap length’ was created to identify sites with extended gaps in monitoring. This histogram helped inform a criterion to exclude 18 additional sites with continuous gaps in sampling of > 14 days (Figure S1). This resulted in a final list of 80 sites across 32 states, all four defined U.S. Census regions (West, South, Midwest, Northeast) and urban and rural areas that covered various population densities to be included in the study (Figure 1) (U.S. Census Bureau 2025).

### 2.2 Calculating *C. auris* Seasonal Detection Rates for Meteorological Seasons

**FIGURE 2:**
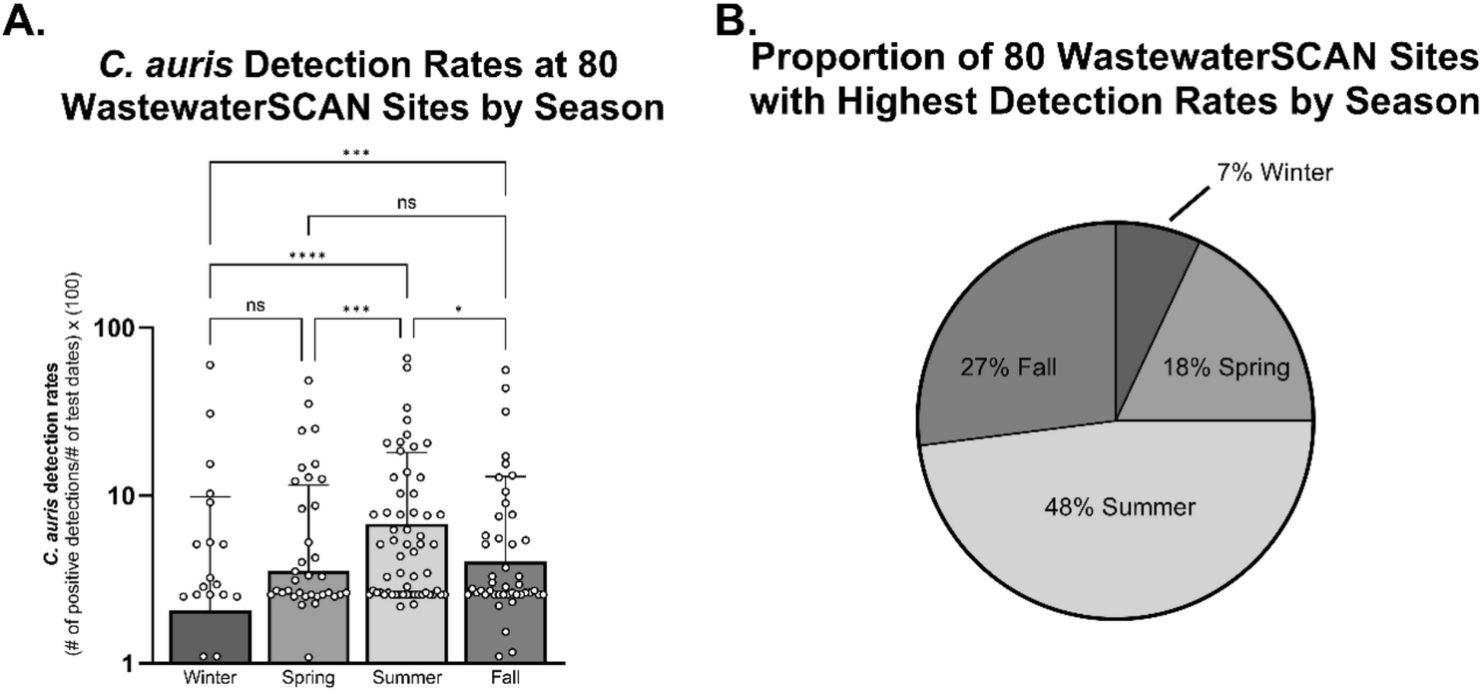
*Candida auris* wastewater seasonal detection rate data at 80 WastewaterSCAN monitoring sites, United States, by meteorological seasons, 12/01/2023 to 11/30/2024. (A) Bars depict mean *C. auris* seasonal detection rates in wastewater for each of the meteorological seasons. White circles represent the individual seasonal detection rates for each site. Non‐detect data are not visible due to logarithmic scale *y*‐axis. Asterisks and associated *p*‐values indicate levels of significance based on Dunn's post hoc test of each possible pairwise seasonal comparisons (*α* = < 0.05, * = *p* < 0.05, *** = *p* < 0.001, **** = *p* < 0.0001, NS = Not Significant). (B) Pie chart shows the proportion of sites that had their highest seasonal detection rates in each season. If a site had its highest seasonal detection rates in multiple seasons, the count was divided equally to each of the tied seasons. For both panels, inclusion criteria required that monitoring sites did not have any gaps in sampling > 14 consecutive days.

Seasonal analysis was performed in collaboration between the CDC and WWSCAN. To prepare data for subsequent *C. auris* detection rate calculations, quantitative results were transformed into binary categorical results of presence and absence (Table S1). Categorical results for each site within the study period were then collated into the meteorological seasons of the northern hemisphere: winter (December, January, February), spring (March, April, May), summer (June, July, August) and fall (September, October, November). *C. auris* seasonal detection rates were calculated for each site by dividing their number of positive detections by their total number of sampling dates within the respective season. Mean distributions of seasonal detection rates across seasons were compared using the non‐parametric Kruskal‐Wallis test followed by a pairwise comparison Dunn's test with Bonferroni correction to compare differences between seasonal pairs using GraphPad Prism 10.0.0 for Windows, GraphPad Software, Boston, Massachusetts US, www.graphpad.com (Figure 2A, Table S2).

### 2.3 Assessing Proportion of Sites With Highest Seasonal Detection Rates Per Season

To assess what proportion of the 80 sites had the highest seasonal detection rate in each season (i.e., spring, summer, fall and winter), sites were classified by the season with the highest seasonal detection rate. If a site had its highest seasonal detection rate in multiple seasons, which occurred at some sites with low detection rates, the count would be divided equally across the tied seasons. For example, if a site had equal seasonal detection rates in both the summer and spring, it would be counted as 0.5 for summer and 0.5 for spring (Table S3). Proportion totals were then divided by total sites analysed and multiplied by 100 to convert into percentages.

### 2.4 Assessing Bootstrapped Probability of Molecular Detection *C. auris* in Wastewater

**FIGURE 3:**
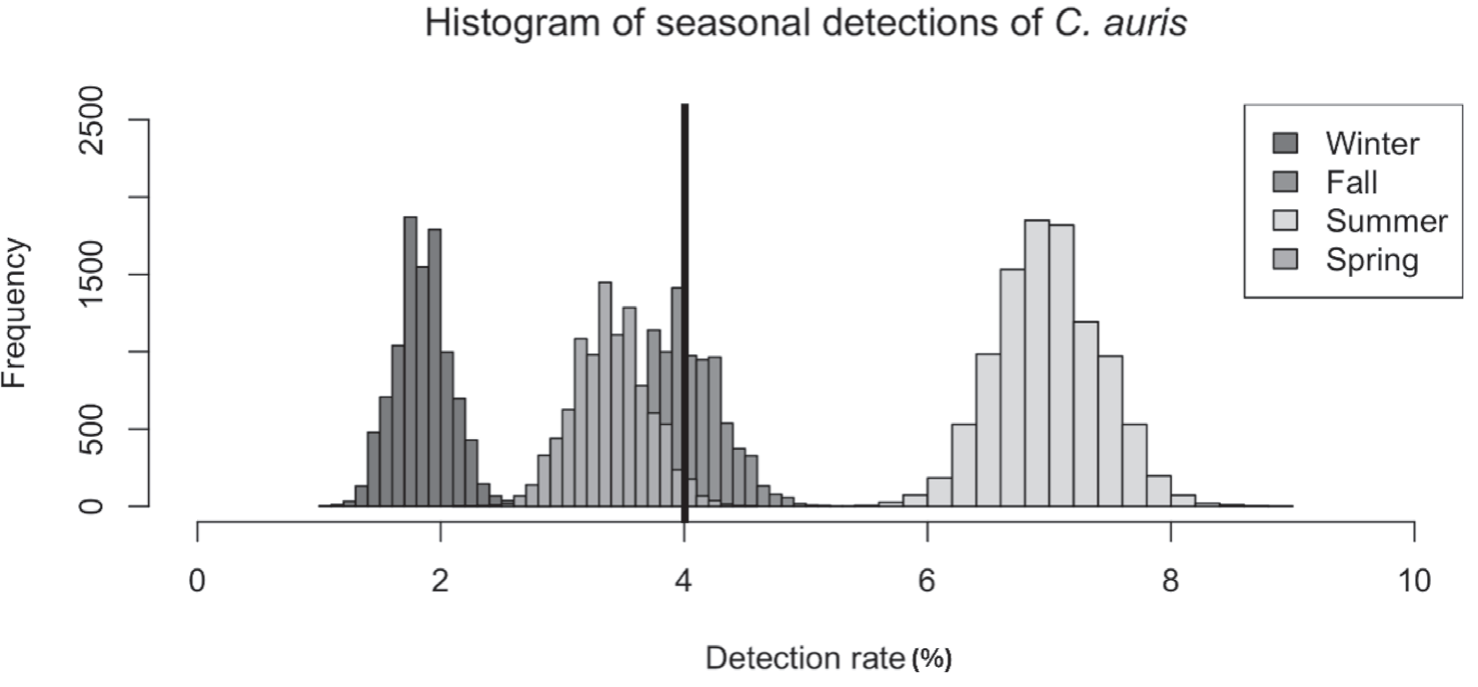
Bootstrapping analysis of seasonal *C. auris* detections comparing 80 WastewaterSCAN monitoring sites across the United States by meteorological seasons 12/01/2023 to 11/30/2024. Each season was bootstrapped 10,000 times, with the resulting frequencies plotted. The black line represents the overall percentage of detections across all samples throughout the year.

We further analysed the seasonality of *C. auris* by analysing the distribution of detections within each season's detection rates. Statistical significance and 95% confidence intervals between the seasons were assessed through bootstrapping (Hesterberg 2011). For each season, a distribution of values was generated through random sampling with replacement 10,000 times (Figure 3). The significance of the difference between seasons was then calculated using the proportion of values below our calculated statistic (i.e., whether the calculated statistics were above the 95% confidence intervals of the generated distribution).

### 2.5 Data Visualisation

Figures were constructed using GraphPad Prism (Version 10, Figure 2, Figures S1 and S2), MapChart (Figure 1), RStudio (Version 5.1, Figure 3), ggplot (Version 3.5.2, Figure 3) and Microsoft PowerPoint (Figure 1).

## 3 Results

### 3.1 *C. auris* Seasonal Detection Rates for Meteorological Seasons

The highest mean seasonal detection rate was in the summer (7%) followed by fall (4%), spring (4%) and winter (2%) (Figure 2A, Table S3). Seasonal detection rates varied significantly across seasons (H = 43.6, *p* < 0.0001, Kruskal‐Wallis test), suggesting a difference in the distribution of detection rates in at least one of the seasons (Figure 2A, Table S2). A Dunn's post hoc test with a Bonferroni correction was performed to identify which seasonal pairs differed significantly, identifying significant differences in seasonal detection rates in winter versus summer (*p* < 0.0001), winter vs. fall (*p* = 0.0008), spring vs. summer (*p* = 0.0004) and summer versus fall (*p* = 0.0477) (Figure 2A, Table S2). Other seasonal comparisons were not significant, including winter versus spring (*p* = 0.0778) and spring versus fall (*p* > 0.9999, Figure 2A, Table S2). If similar more stringent exclusion criteria were used (no day gap in sampling vs. > 14‐day gap), similar results were observed and the interpretation of Kruskal–Wallis test and Dunn's post hoc test remained unchanged (Figure S2B, Table S4).

### 3.2 Proportion of Sites With Highest Seasonal Detection Rates Per Season

Of the 80 WWSCAN sites included in this study, 39 (48%) had the highest *C. auris* seasonal detection rates in the summer, 21 (27%) in the fall, 15 (18%) in the spring and 5 (7%) in the winter (Figure 2B, Table S3). Similar proportions were observed when more stringent exclusion criteria were used (no day gap in sampling vs. > 14‐day gap) (Figure 2B, Figure S1, Figure S2C).

### 3.3 Bootstrap Probability for Seasonal Molecular Detection of *C. auris* in Wastewater

The bootstrapping analysis further demonstrated a significant seasonal pattern (Figure 3). The percentage of detections in winter and summer was found to be significantly different from all other seasons (*p* < 0.01), while there was no significant difference between fall and spring. Winter and summer were also significantly different from the overall distribution (*p* < 0.01).

In summer, 7% of all samples were positive for *C. auris* (95% CI: 6.29, 7.73), spring saw 4% (95% CI: 3.43, 4.54), fall 4% (95% CI: 2.90, 3.92) and winter 2% (95% CI: 1.49, 2.24, Figure 3, Table S1).

## 4 Discussion

This study presents new data documenting seasonal differences in *C. auris w*astewater seasonal detection rates, including a notable increase in the summer, between December 2023—November 2024 across 80 sites nationally. This finding provides valuable context to aid interpretation of future *C. auris* wastewater detections and builds foundational knowledge relevant to current efforts that seek to relate wastewater detections to human cases.

At least two possible mechanisms might explain the observed seasonal differences in *C. auris* seasonal detection rates. The first, is that seasonal trends of human clinical or colonisation case rates could produce corresponding changes in the amount of *C. auris* shed from healthcare settings into wastewater systems. Currently, there are no published reports of seasonal trends in respect to clinical or colonisation cases. One recent study in Baltimore, MD reported an increase in wastewater detections in the spring (Nwaubani et al. 2025). The authors identified a weak, nonsignificant Pearson's correlation between the total number of *C. auris* cases and the number of gene copies found in wastewater. However, it remains unclear whether similar trends will be observed in future years or at other locations. Additional investigations to assess seasonal trends in human *C. auris* clinical or colonisation rates are needed to further test this possible mechanism.

Alternatively, the seasonal changes in detection rates, particularly the increased rates in summer, might be explained by biological amplification of *C. auris* within wastewater systems. *C. auris* growth rates are temperature dependent; the organism grows optimally at 37°C and maintains viability up to 42°C (Rossato and Colombo 2018). Therefore, increased summer detection could be connected to warmer temperatures potentially creating favourable conditions for *C. auris* growth. Another characteristic of *C. auris* is its high tolerance of osmotic stress which when combined with its thermotolerance can contribute to its prolonged survival/viability on both living and non‐living surfaces such as within wastewater systems (Ji‐Seok et al. 2024). Additionally, while WWSCAN only measures *C. auris* nucleic‐acid concentrations, viable *C. auris* has been cultured from a community wastewater site representing the output of over 1 million residents in Nevada (Rossi et al. 2023). Whole genome sequencing revealed that the Nevada isolate recovered was highly related to isolates causing local outbreaks. This observation establishes that *C. auris* can disseminate from healthcare settings and persist downstream in wastewater systems.

Considering the thermotolerance and persistence of *C. auris* , it seems plausible that *C. auris* may also grow in a nutrient‐rich substrate such as wastewater. This possibility raises concern about the potential for wastewater to serve as a reservoir for pathogen amplification and dissemination, which would complicate interpretation of wastewater detection events. Whether *C. auris* can grow in wastewater has not been experimentally confirmed. This hypothesis could be tested by performing growth experiments in various wastewater conditions as a proof of concept. Similarly, better understanding whether other microbial pathogens can amplify in wastewater would be of value to the field.

This study has several limitations. First, the 80 WWSCAN sites included in this study are not evenly distributed across the United States, resulting in variability in how well some regions are represented compared to others (Figure 1). For this reason, the ability to cross‐compare geographical regions and understand differences based on case burden and local epidemiology was limited. Second, sampling schedules were not consistent across the sites, with sampling frequencies ranging from twice weekly to daily, though we do not believe that this variation in sampling frequency affected key findings. Third, our data set does not include culture‐based testing for *C. auris* , therefore cannot be distinguished from non‐viable organisms.

Future work of interest could explore relevant temperature and weather data sets, which could help explain the differences in seasonal detection rates observed. For example, rainfall conditions could affect seasonal detection rates from wastewater facilities that share sanitary wastewater and stormwater systems due to higher flow rates. A recent model predicted that detection rates could decrease from facilities with higher flow rates caused by higher population coverage (Chavez et al. 2024). Further efforts to relate the data presented here with respective weather data sets are warranted and may help clarify the underlying mechanisms driving the changes in seasonal detection rates observed.

In summary, this work documents changes in *C. auris* wastewater seasonal detection rates across seasons, with a notable increase in the summer. This work contributes to the understanding of *C. auris* wastewater by highlighting seasonal patterns with pertinence to ongoing efforts to apply wastewater surveillance to inform public health decisions.

## Author Contributions

**Jorge Chavez:** conceptualization, methodology, visualization, writing – review and editing, data curation, writing – original draft, formal analysis, investigation, validation. **Alessandro Zulli:** investigation, methodology, data curation, writing – review and editing, visualization, formal analysis, validation, conceptualization. **Dustin Duffy:** data curation, formal analysis, methodology, writing – review and editing, investigation. **Mariya Campbell:** data curation, writing – review and editing, investigation, validation. **Meghan Lyman:** writing – review and editing, conceptualization, data curation. **Angela Coulliette‐Salmond:** supervision, writing – review and editing, data curation, investigation, validation. **D. Joseph Sexton:** writing – review and editing, writing – original draft, conceptualization, methodology, supervision, investigation, visualization.

## Funding

This work was supported by the Oak Ridge Institute for Science and Education and the Sergey Brin Family Foundation.

## Ethics Statement

All reported work in this study is original. The content and authorship of the submitted manuscript have been approved by all authors and that all prevailing local, national and international regulations and conventions and normal scientific ethical practices have been respected.

## Consent

The authors have nothing to report.

## Conflicts of Interest

The authors declare no conflicts of interest.

## Supporting information


**Figure S1:** emi470376‐sup‐0001‐supinfo.docx. *Candida auris* sampling gap exclusion data at 80 WastewaterSCAN sites across the United States by meteorological seasons 12/01/2023 to 11/30/2024.
**Figure S2:** *Candida auris* wastewater seasonal detection rate and sampling gap exclusion data at 98 WastewaterSCAN monitoring sites across the United States by meteorological seasons 12/01/2023 to 11/30/2024.
**Table S1:**. Seasonal binary categorical positive and negative *C. auris* detections from 80 WastewaterSCAN monitoring sites across the United States 12/01/2023 to 11/30/2024 without consecutive sampling gaps > 14 days
**Table S2:** Dunn's test seasonal pairs comparison of *C. auris* detection rates from 80 WastewaterSCAN monitoring sites across the United States 12/01/2023 to 11/30/2024 without consecutive sampling gaps > 14 days. (* = *p* < 0.05, ** = *p* < 0.01, *** = *p* < 0.001, **** = *p* < 0.0001, NS = Not Significant).
**Table S3:** Seasonal Proportion of 80 WastewaterSCAN monitoring sites across the United States with highest *C. auris* detection rates 12/01/2023 to 11/30/2024 without consecutive sampling gaps > 14 days. The grey cells represent the season with highest detection rate.
**Table S4:** Dunn's test seasonal pairs comparison of *C. auris* seasonal detection rates from 98 WastewaterSCAN monitoring sites across the United States 12/01/2023 to 11/30/2024 without consecutive sampling gaps exclusion (* = *p* < 0.05, ** = *p* < 0.01, *** = *p* < 0.001, **** = *p* < 0.0001, NS = Not Significant).

## Data Availability

The data that supports the findings of this study are available in the Supporting Information of this article.
