# Supplementary material for: Seasonal Analysis of Candida auris Detection Rates at 80 Wastewater Monitoring Sites in the United States, December 2023–November 2024
Source: Environ Microbiol Rep. 2026 Jun 8;18(3):e70376. doi: 10.1111/1758-2229.70376 (PMC13246258; doi:10.1111/1758-2229.70376)
Supplement: Supplementary file 1 — Figure S1: emi470376‐sup‐0001‐supinfo.docx. Candida auris sampling gap exclusion data at 80 WastewaterSCAN sites across the United States by meteorological seasons 12/01/2023 to 11/30/2024. Figure S2: Candida auris wastewater seasonal detection rate and sampling gap exclusion data at 98 WastewaterSCAN monitoring sites across the United States by meteorological seasons 12/01/2023 to 11/30/2024. Table S1:. Seasonal binary categorical positive and negative C. auris detections from 80 WastewaterSCAN monitoring sites across the United States 12/01/2023 to 11/30/2024 without consecutive sampling gaps > 14 days Table S2: Dunn's test seasonal pairs comparison of C. auris detection rates from 80 WastewaterSCAN monitoring sites across the United States 12/01/2023 to 11/30/2024 without consecutive sampling gaps > 14 days. (* = p < 0.05, ** = p < 0.01, *** = p < 0.001, **** = p < 0.0001, NS = Not Significant). Table S3: Seasonal Proportion of 80 WastewaterSCAN monitoring sites across the United States with highest C. auris detection rates 12/01/2023 to 11/30/2024 without consecutive sampling gaps > 14 days. The grey cells represent the season with highest detection rate. Table S4: Dunn's test seasonal pairs comparison of C. auris seasonal detection rates from 98 WastewaterSCAN monitoring sites across the United States 12/01/2023 to 11/30/2024 without consecutive sampling gaps exclusion (* = p < 0.05, ** = p < 0.01, *** = p < 0.001, **** = p < 0.0001, NS = Not Significant). [file EMI4-18-e70376-s001.docx]

**Supplemental**

**
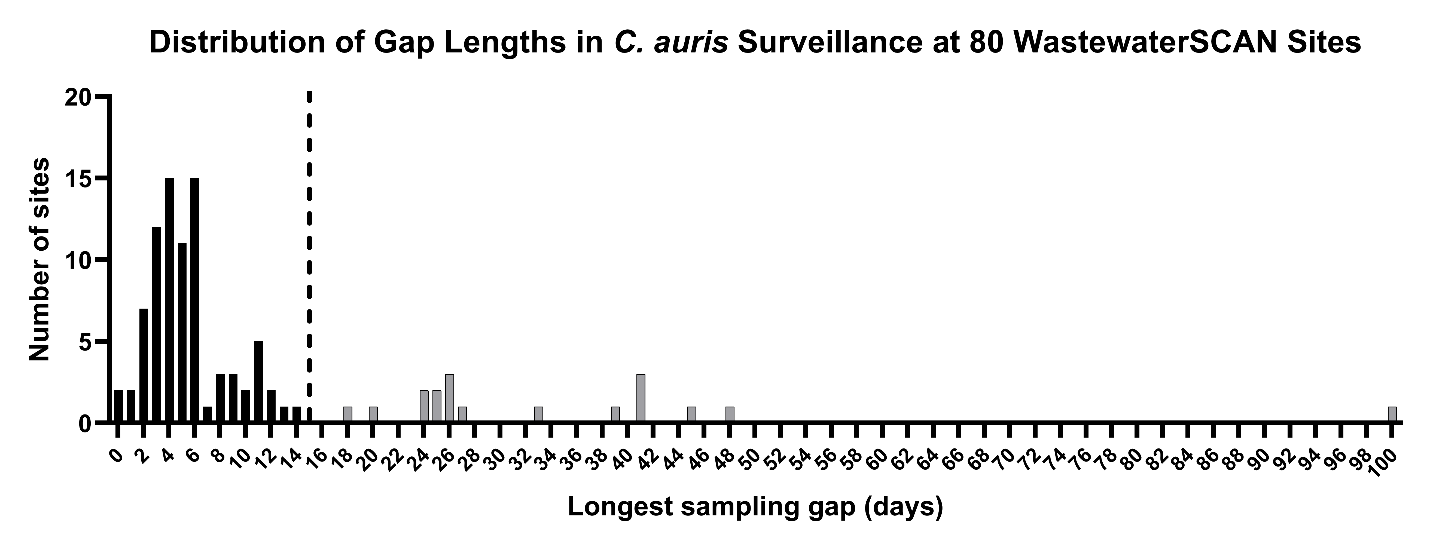
Figure S1. *Candida auris* sampling gap exclusion data at 80 WastewaterSCAN sites across the United States by meteorological seasons 12/01/2023 to 11/30/2024**

Histogram of gaps in sampling used to determine exclusion criteria, highlighting a drop-off in sampling of > 14 days. Study inclusion criteria: sites sampled 12/01/2023 to 11/30/2024, sites with collections throughout all meteorological seasons (winter, spring, summer, fall), sites without consecutive sampling gaps > 14 days.


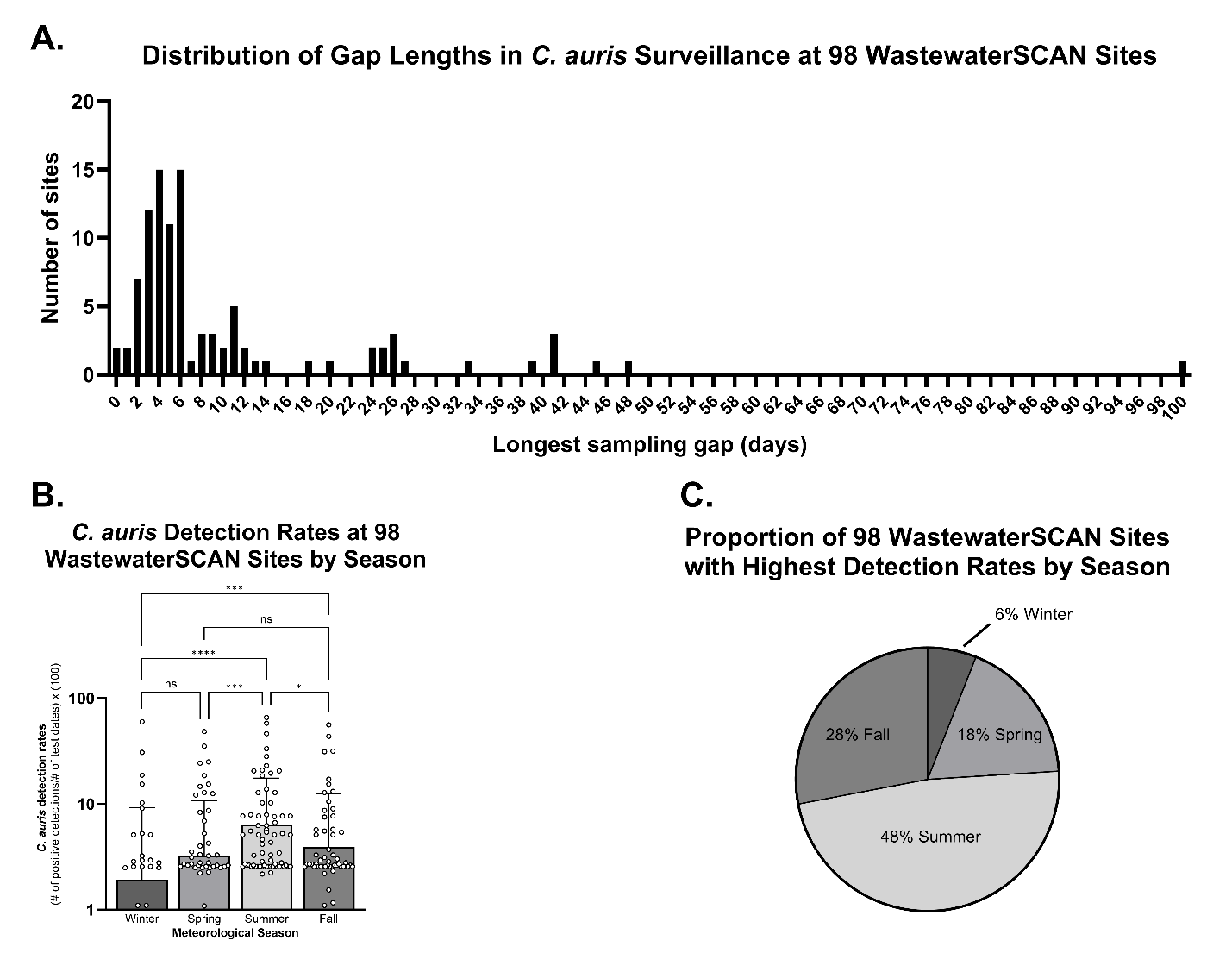
**Figure S2. *Candida auris* wastewater seasonal detection rate and sampling gap exclusion data at 98 WastewaterSCAN monitoring sites across the United States by meteorological seasons 12/01/2023 to 11/30/2024**

**A.** Histogram of gaps in sampling used to determine exclusion criteria, highlighting no sampling gap exclusion. **B.** Bars depict mean *C. auris* seasonal detection rates in wastewater for each of the meteorological seasons, winter, spring, summer, and fall. White circles represent the individual detection rates for each site. Non-detect data is not visible due to logarithmic scale y-axis. Asterisks and associated P-values indicate levels of significance based on Dunn’s post-hoc test of each possible pairwise seasonal comparison (* = *p* < 0.05, ** = *p* < 0.01, *** = *p* < 0.001, **** = *p* < 0.0001, NS = Not Significant). **C.** Pie chart shows the proportion of sites that had their highest seasonal detection rates in each season. If a site had its highest seasonal detection rate in multiple seasons, the count was divided equally among each of the tied seasons.

**Table S1.** **Seasonal** **binary categorical positive and negative *C. auris* detections from 80 WastewaterSCAN monitoring sites across the United States 12/01/2023 to 11/30/2024 without consecutive sampling gaps > 14 days**

|  | **Winter** | | | | **Spring** | | | **Summer** | | | **Fall** | | | |
| --- | --- | --- | --- | --- | --- | --- | --- | --- | --- | --- | --- | --- | --- | --- |
| **Site** | **positive** | **negative** | **days** **sampled** | **positive** | | **negative** | **days** **samples** | **positive** | **negative** | **days**  **sampled** | **positive** | | **negative** | **days** **sampled** |
| **Site 1** | 0 | 39 | 39 | 1 | | 39 | 40 | 2 | 37 | 39 | | 1 | 38 | 39 |
| **Site 2** | 0 | 39 | 39 | 0 | | 39 | 39 | 3 | 36 | 39 | | 1 | 38 | 39 |
| **Site 3** | 3 | 30 | 33 | 0 | | 37 | 37 | 1 | 28 | 29 | | 0 | 36 | 36 |
| **Site 4** | 0 | 39 | 39 | 0 | | 40 | 40 | 1 | 38 | 39 | | 0 | 39 | 39 |
| **Site 5** | 0 | 35 | 35 | 1 | | 39 | 40 | 0 | 38 | 38 | | 1 | 36 | 37 |
| **Site 6** | 0 | 91 | 91 | 1 | | 91 | 92 | 2 | 90 | 92 | | 3 | 88 | 91 |
| **Site 7** | 1 | 38 | 39 | 1 | | 39 | 40 | 1 | 38 | 39 | | 1 | 38 | 39 |
| **Site 8** | 0 | 39 | 39 | 1 | | 38 | 39 | 1 | 38 | 39 | | 2 | 37 | 39 |
| **Site 9** | 0 | 35 | 35 | 1 | | 36 | 37 | 0 | 38 | 38 | | 1 | 33 | 34 |
| **Site 10** | 0 | 39 | 39 | 0 | | 40 | 40 | 1 | 38 | 39 | | 0 | 39 | 39 |
| **Site 11** | 0 | 39 | 39 | 0 | | 40 | 40 | 1 | 38 | 39 | | 1 | 37 | 38 |
| **Site 12** | 0 | 84 | 84 | 3 | | 82 | 85 | 25 | 64 | 89 | | 8 | 81 | 89 |
| **Site 13** | 0 | 38 | 38 | 6 | | 33 | 39 | 3 | 35 | 38 | | 0 | 36 | 36 |
| **Site 14** | 1 | 90 | 91 | 8 | | 84 | 92 | 18 | 74 | 92 | | 2 | 89 | 91 |
| **Site 15** | 0 | 38 | 38 | 0 | | 39 | 39 | 1 | 37 | 38 | | 1 | 37 | 38 |
| **Site 16** | 0 | 91 | 91 | 0 | | 68 | 68 | 3 | 62 | 65 | | 1 | 64 | 65 |
| **Site 17** | 1 | 90 | 91 | 3 | | 88 | 91 | 3 | 89 | 92 | | 0 | 91 | 91 |
| **Site 18** | 0 | 91 | 91 | 0 | | 92 | 92 | 3 | 88 | 91 | | 3 | 88 | 91 |
| **Site 19** | 0 | 22 | 22 | 0 | | 24 | 24 | 1 | 22 | 23 | | 0 | 23 | 23 |
| **Site 20** | 0 | 38 | 38 | 0 | | 38 | 38 | 4 | 35 | 39 | | 2 | 35 | 37 |
| **Site 21** | 0 | 90 | 90 | 3 | | 87 | 90 | 2 | 87 | 89 | | 1 | 85 | 86 |
| **Site 22** | 0 | 84 | 84 | 2 | | 86 | 88 | 7 | 85 | 92 | | 1 | 90 | 91 |
| **Site 23** | 0 | 40 | 40 | 0 | | 39 | 39 | 0 | 39 | 39 | | 1 | 38 | 39 |
| **Site 24** | 0 | 39 | 39 | 0 | | 40 | 40 | 0 | 39 | 39 | | 1 | 38 | 39 |
| **Site 25** | 0 | 33 | 33 | 0 | | 31 | 31 | 2 | 30 | 32 | | 0 | 26 | 26 |
| **Site 26** | 0 | 36 | 36 | 0 | | 38 | 38 | 1 | 37 | 38 | | 0 | 32 | 32 |
| **Site 27** | 0 | 37 | 37 | 1 | | 38 | 39 | 1 | 38 | 39 | | 0 | 33 | 33 |
| **Site 28** | 0 | 29 | 29 | 0 | | 31 | 31 | 2 | 30 | 32 | | 1 | 26 | 27 |
| **Site 29** | 1 | 39 | 40 | 1 | | 36 | 37 | 0 | 37 | 37 | | 0 | 39 | 39 |
| **Site 30** | 0 | 39 | 39 | 0 | | 38 | 38 | 1 | 38 | 39 | | 0 | 39 | 39 |
| **Site 31** | 0 | 37 | 37 | 0 | | 40 | 40 | 1 | 38 | 39 | | 0 | 39 | 39 |
| **Site 32** | 0 | 39 | 39 | 0 | | 38 | 38 | 8 | 31 | 39 | | 4 | 34 | 38 |
| **Site 33** | 0 | 42 | 42 | 2 | | 45 | 47 | 0 | 49 | 49 | | 1 | 42 | 43 |
| **Site 34** | 0 | 39 | 39 | 0 | | 41 | 41 | 4 | 35 | 39 | | 0 | 39 | 39 |
| **Site 35** | 1 | 38 | 39 | 1 | | 38 | 39 | 2 | 37 | 39 | | 0 | 33 | 33 |
| **Site 36** | 0 | 39 | 39 | 0 | | 39 | 39 | 3 | 35 | 38 | | 1 | 37 | 38 |
| **Site 37** | 0 | 39 | 39 | 0 | | 40 | 40 | 1 | 38 | 39 | | 1 | 38 | 39 |
| **Site 38** | 0 | 36 | 36 | 0 | | 38 | 38 | 1 | 38 | 39 | | 0 | 38 | 38 |
| **Site 39** | 0 | 30 | 30 | 0 | | 38 | 38 | 0 | 37 | 37 | | 1 | 35 | 36 |
| **Site 40** | 21 | 14 | 35 | 14 | | 15 | 29 | 4 | 25 | 29 | | 6 | 29 | 35 |
| **Site 41** | 0 | 39 | 39 | 1 | | 39 | 40 | 0 | 39 | 39 | | 0 | 39 | 39 |
| **Site 42** | 2 | 37 | 39 | 0 | | 40 | 40 | 0 | 39 | 39 | | 0 | 39 | 39 |
| **Site 43** | 1 | 33 | 34 | 0 | | 38 | 38 | 1 | 36 | 37 | | 0 | 34 | 34 |
| **Site 44** | 6 | 33 | 39 | 3 | | 33 | 36 | 0 | 36 | 36 | | 5 | 33 | 38 |
| **Site 45** | 0 | 39 | 39 | 0 | | 40 | 40 | 0 | 39 | 39 | | 1 | 36 | 37 |
| **Site 46** | 0 | 40 | 40 | 1 | | 37 | 38 | 1 | 38 | 39 | | 0 | 39 | 39 |
| **Site 47** | 0 | 39 | 39 | 5 | | 34 | 39 | 7 | 31 | 38 | | 3 | 37 | 40 |
| **Site 48** | 0 | 35 | 35 | 1 | | 37 | 38 | 0 | 38 | 38 | | 0 | 38 | 38 |
| **Site 49** | 0 | 38 | 38 | 0 | | 37 | 37 | 3 | 36 | 39 | | 0 | 38 | 38 |
| **Site 50** | 1 | 30 | 31 | 0 | | 31 | 31 | 2 | 33 | 35 | | 0 | 30 | 30 |
| **Site 51** | 0 | 40 | 40 | 0 | | 39 | 39 | 2 | 37 | 39 | | 0 | 38 | 38 |
| **Site 52** | 0 | 38 | 38 | 0 | | 39 | 39 | 1 | 38 | 39 | | 1 | 36 | 37 |
| **Site 53** | 0 | 39 | 39 | 0 | | 40 | 40 | 1 | 38 | 39 | | 1 | 37 | 38 |
| **Site 54** | 0 | 39 | 39 | 0 | | 40 | 40 | 2 | 37 | 39 | | 0 | 38 | 38 |
| **Site 55** | 2 | 37 | 39 | 6 | | 35 | 41 | 5 | 34 | 39 | | 3 | 49 | 52 |
| **Site 56** | 1 | 34 | 35 | 0 | | 38 | 38 | 0 | 39 | 39 | | 1 | 32 | 33 |
| **Site 57** | 0 | 35 | 35 | 0 | | 37 | 37 | 2 | 35 | 37 | | 1 | 34 | 35 |
| **Site 58** | 0 | 38 | 38 | 0 | | 39 | 39 | 1 | 36 | 37 | | 0 | 38 | 38 |
| **Site 59** | 0 | 33 | 33 | 2 | | 36 | 38 | 1 | 34 | 35 | | 0 | 35 | 35 |
| **Site 60** | 0 | 25 | 25 | 1 | | 31 | 32 | 1 | 28 | 29 | | 0 | 34 | 34 |
| **Site 61** | 0 | 26 | 26 | 0 | | 30 | 30 | 1 | 37 | 38 | | 1 | 38 | 39 |
| **Site 62** | 4 | 35 | 39 | 10 | | 30 | 40 | 9 | 30 | 39 | | 3 | 36 | 39 |
| **Site 63** | 12 | 27 | 39 | 9 | | 28 | 37 | 25 | 13 | 38 | | 12 | 26 | 38 |
| **Site 64** | 0 | 37 | 37 | 5 | | 35 | 40 | 13 | 26 | 39 | | 17 | 22 | 39 |
| **Site 65** | 1 | 39 | 40 | 0 | | 34 | 34 | 1 | 38 | 39 | | 2 | 37 | 39 |
| **Site 66** | 0 | 39 | 39 | 0 | | 39 | 39 | 1 | 38 | 39 | | 0 | 39 | 39 |
| **Site 67** | 0 | 34 | 34 | 0 | | 39 | 39 | 0 | 37 | 37 | | 2 | 34 | 36 |
| **Site 68** | 0 | 34 | 34 | 1 | | 37 | 38 | 0 | 37 | 37 | | 0 | 37 | 37 |
| **Site 69** | 0 | 36 | 36 | 0 | | 35 | 35 | 0 | 37 | 37 | | 1 | 38 | 39 |
| **Site 70** | 0 | 35 | 35 | 0 | | 38 | 38 | 0 | 38 | 38 | | 6 | 33 | 39 |
| **Site 71** | 0 | 39 | 39 | 0 | | 38 | 38 | 0 | 39 | 39 | | 1 | 38 | 39 |
| **Site 72** | 0 | 37 | 37 | 1 | | 44 | 45 | 5 | 34 | 39 | | 5 | 34 | 39 |
| **Site 73** | 0 | 33 | 33 | 0 | | 37 | 37 | 1 | 37 | 38 | | 0 | 35 | 35 |
| **Site 74** | 2 | 36 | 38 | 13 | | 24 | 37 | 22 | 16 | 38 | | 19 | 15 | 34 |
| **Site 75** | 0 | 39 | 39 | 0 | | 40 | 40 | 3 | 36 | 39 | | 0 | 38 | 38 |
| **Site 76** | 0 | 25 | 25 | 1 | | 24 | 25 | 5 | 19 | 24 | | 0 | 26 | 26 |
| **Site 77** | 0 | 37 | 37 | 1 | | 37 | 38 | 0 | 37 | 37 | | 0 | 37 | 37 |
| **Site 78** | 0 | 39 | 39 | 1 | | 38 | 39 | 0 | 39 | 39 | | 0 | 38 | 38 |
| **Site 79** | 0 | 39 | 39 | 0 | | 40 | 40 | 0 | 39 | 39 | | 1 | 38 | 39 |
| **Site 80** | 1 | 38 | 39 | 4 | | 29 | 33 | 8 | 31 | 39 | | 0 | 39 | 39 |
| **Total** | 62 | 3289 | 3351 | 116 | | 3298 | 3414 | 238 | 3165 | 3403 | | 133 | 3225 | 3358 |
| **% Total** | 2% | 98.15% |  | 4% | | 96.60% |  | 7% | 93.01% |  | | 4% | 96.04% |  |

**Table S2. Dunn’s test seasonal pairs comparison of *C. auris* detection rates from 80 WastewaterSCAN monitoring sites across the United States 12/01/2023 to 11/30/2024 without consecutive sampling gaps > 14 days.** (* = *p* < 0.05, ** = *p* < 0.01, *** = *p* < 0.001, **** = *p* < 0.0001, NS = Not Significant)

| **Dunn's multiple comparisons test** | **Summary** | **Adjusted P value** |
| --- | --- | --- |
| **Winter vs. Spring** | ns | 0.077832 |
| **Winter vs. Summer** | **** | <0.000001 |
| **Winter vs. Fall** | *** | 0.000814 |
| **Spring vs. Summer** | *** | 0.000404 |
| **Spring vs. Fall** | ns | >0.999999 |
| **Summer vs. Fall** | * | 0.047679 |

**Table S3. Seasonal Proportion of 80 WastewaterSCAN monitoring sites across the United States with highest *C. auris* detection rates 12/01/2023 to 11/30/2024 without consecutive sampling gaps > 14 days.** The **g**ray cells represent the season with highest detection rate

|  | **Seasonal detection rates** | | | | **Highest rate portioned values** | | | |
| --- | --- | --- | --- | --- | --- | --- | --- | --- |
| **Site** | **Winter** | **Spring** | **Summer** | **Fall** | **Winter** | **Spring** | **Summer** | **Fall** |
| **Site 1** | 0.00 | 2.50 | 5.13 | 2.56 |  |  | 1 |  |
| **Site 2** | 0.00 | 0.00 | 7.69 | 2.56 |  |  | 1 |  |
| **Site 3** | 9.09 | 0.00 | 3.45 | 0.00 | 1 |  |  |  |
| **Site 4** | 0.00 | 0.00 | 2.56 | 0.00 |  |  | 1 |  |
| **Site 5** | 0.00 | 2.50 | 0.00 | 2.70 |  |  |  | 1 |
| **Site 6** | 0.00 | 1.09 | 2.17 | 3.30 |  |  |  | 1 |
| **Site 7** | 2.56 | 2.50 | 2.56 | 2.56 | 0.33 |  | 0.33 | 0.33 |
| **Site 8** | 0.00 | 2.56 | 2.56 | 5.13 |  |  |  | 1 |
| **Site 9** | 0.00 | 2.70 | 0.00 | 2.94 |  |  |  | 1 |
| **Site 10** | 0.00 | 0.00 | 2.56 | 0.00 |  |  | 1 |  |
| **Site 11** | 0.00 | 0.00 | 2.56 | 2.63 |  |  |  | 1 |
| **Site 12** | 0.00 | 3.53 | 28.09 | 8.99 |  |  | 1 |  |
| **Site 13** | 0.00 | 15.38 | 7.89 | 0.00 |  | 1 |  |  |
| **Site 14** | 1.10 | 8.70 | 19.57 | 2.20 |  |  | 1 |  |
| **Site 15** | 0.00 | 0.00 | 2.63 | 2.63 |  |  | 0.5 | 0.5 |
| **Site 16** | 0.00 | 0.00 | 4.62 | 1.54 |  |  | 1 |  |
| **Site 17** | 1.10 | 3.30 | 3.26 | 0.00 |  | 1 |  |  |
| **Site 18** | 0.00 | 0.00 | 3.30 | 3.30 |  |  | 0.5 | 0.5 |
| **Site 19** | 0.00 | 0.00 | 4.35 | 0.00 |  |  | 1 |  |
| **Site 20** | 0.00 | 0.00 | 10.26 | 5.41 |  |  | 1 |  |
| **Site 21** | 0.00 | 3.33 | 2.25 | 1.16 |  | 1 |  |  |
| **Site 22** | 0.00 | 2.27 | 7.61 | 1.10 |  |  | 1 |  |
| **Site 23** | 0.00 | 0.00 | 0.00 | 2.56 |  |  |  | 1 |
| **Site 24** | 0.00 | 0.00 | 0.00 | 2.56 |  |  |  | 1 |
| **Site 25** | 0.00 | 0.00 | 6.25 | 0.00 |  |  | 1 |  |
| **Site 26** | 0.00 | 0.00 | 2.63 | 0.00 |  |  | 1 |  |
| **Site 27** | 0.00 | 2.56 | 2.56 | 0.00 |  | 0.5 | 0.5 |  |
| **Site 28** | 0.00 | 0.00 | 6.25 | 3.70 |  |  | 1 |  |
| **Site 29** | 2.50 | 2.70 | 0.00 | 0.00 |  | 1 |  |  |
| **Site 30** | 0.00 | 0.00 | 2.56 | 0.00 |  |  | 1 |  |
| **Site 31** | 0.00 | 0.00 | 2.56 | 0.00 |  |  | 1 |  |
| **Site 32** | 0.00 | 0.00 | 20.51 | 10.53 |  |  | 1 |  |
| **Site 33** | 0.00 | 4.26 | 0.00 | 2.33 |  | 1 |  |  |
| **Site 34** | 0.00 | 0.00 | 10.26 | 0.00 |  |  | 1 |  |
| **Site 35** | 2.56 | 2.56 | 5.13 | 0.00 |  |  | 1 |  |
| **Site 36** | 0.00 | 0.00 | 7.89 | 2.63 |  |  | 1 |  |
| **Site 37** | 0.00 | 0.00 | 2.56 | 2.56 |  |  | 0.5 | 0.5 |
| **Site 38** | 0.00 | 0.00 | 2.56 | 0.00 |  |  | 1 |  |
| **Site 39** | 0.00 | 0.00 | 0.00 | 2.78 |  |  |  | 1 |
| **Site 40** | 60.00 | 48.28 | 13.79 | 17.14 | 1 |  |  |  |
| **Site 41** | 0.00 | 2.50 | 0.00 | 0.00 |  | 1 |  |  |
| **Site 42** | 5.13 | 0.00 | 0.00 | 0.00 | 1 |  |  |  |
| **Site 43** | 2.94 | 0.00 | 2.70 | 0.00 | 1 |  |  |  |
| **Site 44** | 15.38 | 8.33 | 0.00 | 13.16 | 1 |  |  |  |
| **Site 45** | 0.00 | 0.00 | 0.00 | 2.70 |  |  |  | 1 |
| **Site 46** | 0.00 | 2.63 | 2.56 | 0.00 |  | 1 |  |  |
| **Site 47** | 0.00 | 12.82 | 18.42 | 7.50 |  |  | 1 |  |
| **Site 48** | 0.00 | 2.63 | 0.00 | 0.00 |  | 1 |  |  |
| **Site 49** | 0.00 | 0.00 | 7.69 | 0.00 |  |  | 1 |  |
| **Site 50** | 3.23 | 0.00 | 5.71 | 0.00 |  |  | 1 |  |
| **Site 51** | 0.00 | 0.00 | 5.13 | 0.00 |  |  | 1 |  |
| **Site 52** | 0.00 | 0.00 | 2.56 | 2.70 |  |  |  | 1 |
| **Site 53** | 0.00 | 0.00 | 2.56 | 2.63 |  |  |  | 1 |
| **Site 54** | 0.00 | 0.00 | 5.13 | 0.00 |  |  | 1 |  |
| **Site 55** | 5.13 | 14.63 | 12.82 | 5.77 |  | 1 |  |  |
| **Site 56** | 2.86 | 0.00 | 0.00 | 3.03 |  |  |  | 1 |
| **Site 57** | 0.00 | 0.00 | 5.41 | 2.86 |  |  | 1 |  |
| **Site 58** | 0.00 | 0.00 | 2.70 | 0.00 |  |  | 1 |  |
| **Site 59** | 0.00 | 5.26 | 2.86 | 0.00 |  | 1 |  |  |
| **Site 60** | 0.00 | 3.13 | 3.45 | 0.00 |  |  | 1 |  |
| **Site 61** | 0.00 | 0.00 | 2.63 | 2.56 |  |  | 1 |  |
| **Site 62** | 10.26 | 25.00 | 23.08 | 7.69 |  | 1 |  |  |
| **Site 63** | 30.77 | 24.32 | 65.79 | 31.58 |  |  | 1 |  |
| **Site 64** | 0.00 | 12.50 | 33.33 | 43.59 |  |  |  | 1 |
| **Site 65** | 2.50 | 0.00 | 2.56 | 5.13 |  |  |  | 1 |
| **Site 66** | 0.00 | 0.00 | 2.56 | 0.00 |  |  | 1 |  |
| **Site 67** | 0.00 | 0.00 | 0.00 | 5.56 |  |  |  | 1 |
| **Site 68** | 0.00 | 2.63 | 0.00 | 0.00 |  | 1 |  |  |
| **Site 69** | 0.00 | 0.00 | 0.00 | 2.56 |  |  |  | 1 |
| **Site 70** | 0.00 | 0.00 | 0.00 | 15.38 |  |  |  | 1 |
| **Site 71** | 0.00 | 0.00 | 0.00 | 2.56 |  |  |  | 1 |
| **Site 72** | 0.00 | 2.22 | 12.82 | 12.82 |  |  | 0.5 | 0.5 |
| **Site 73** | 0.00 | 0.00 | 2.63 | 0.00 |  |  | 1 |  |
| **Site 74** | 5.26 | 35.14 | 57.89 | 55.88 |  |  | 1 |  |
| **Site 75** | 0.00 | 0.00 | 7.69 | 0.00 |  |  | 1 |  |
| **Site 76** | 0.00 | 4.00 | 20.83 | 0.00 |  |  | 1 |  |
| **Site 77** | 0.00 | 2.63 | 0.00 | 0.00 |  | 1 |  |  |
| **Site 78** | 0.00 | 2.56 | 0.00 | 0.00 |  | 1 |  |  |
| **Site 79** | 0.00 | 0.00 | 0.00 | 2.56 |  |  |  | 1 |
| **Site 80** | 2.56 | 12.12 | 20.51 | 0.00 |  |  | 1 |  |
| **Mode** | 0.00 | 0.00 | 0.00 | 0.00 |  |  |  |  |
| **Min** | 0.00 | 0.00 | 0.00 | 0.00 |  |  |  |  |
| **Max** | 60.00 | 48.28 | 65.79 | 55.88 |  |  |  |  |
| **StDEV** | 7.77 | 7.98 | 11.26 | 8.87 |  |  |  |  |
| **Mean Rate** | 0.02 | 0.04 | 0.07 | 0.04 |  |  |  |  |
| **Mean Rate %** | 2 | 4 | 7 | 4 |  |  |  |  |
| **Proportion Total** |  |  |  |  | 5.3 | 14.5 | 38.8 | 21.3 |
| **Proportion %** |  |  |  |  | 7% | 18% | 48% | 27% |

**Table S4. Dunn’s test seasonal pairs comparison of *C. auris* seasonal detection rates from 98 WastewaterSCAN monitoring sites across the United States 12/01/2023 to 11/30/2024 without consecutive sampling gaps exclusion** (* = *p* < 0.05, ** = *p* < 0.01, *** = *p* < 0.001, **** = *p* < 0.0001, NS = Not Significant)

| **Dunn's multiple comparisons test** | **Summary** | **Adjusted P value** |
| --- | --- | --- |
| **Winter vs. Spring** | ns | 0.068212 |
| **Winter vs. Summer** | **** | <0.000001 |
| **Winter vs. Fall** | *** | 0.000265 |
| **Spring vs. Summer** | *** | 0.000157 |
| **Spring vs. Fall** | ns | 0.722283 |
| **Summer vs. Fall** | * | 0.048133 |
